# All-optical Reflection-mode Microscopic Histology of Unstained Human Tissues

**DOI:** 10.1038/s41598-019-49849-9

**Published:** 2019-09-16

**Authors:** Saad Abbasi, Martin Le, Bazil Sonier, Deepak Dinakaran, Gilbert Bigras, Kevan Bell, John R. Mackey, Parsin Haji Reza

**Affiliations:** 1 PhotoMedicine Labs, Department of Systems Design Engineering, University of Waterloo, Waterloo, Ontario N2L 3G1 Canada; 2 illumiSonics, Inc., Department of Systems Design Engineering, University of Waterloo, Waterloo, Ontario N2L 3G1 Canada; 3 Department of Oncology, University of Alberta, Edmonton, Alberta T6G 2V1 Canada; 4 Department of Laboratory Medicine and Pathology, University of Alberta, Edmonton, Alberta T6G 2V1 Canada

**Keywords:** Biomedical engineering, Applied optics

## Abstract

Surgical oncologists depend heavily on visual field acuity during cancer resection surgeries for *in-situ* margin assessment. Clinicians must wait up to two weeks for results from a pathology lab to confirm a post-operative diagnosis, potentially resulting in subsequent treatments. Currently, there are no clinical tools that can visualize diagnostically pertinent tissue information *in-situ*. Here, we present the first microscopy capable of non-contact label-free visualization of human cellular morphology in a reflection-mode apparatus. This is possible with the recently reported imaging modality called photoacoustic remote sensing microscopy which enables non-contact detection of optical absorption contrast. By taking advantage of the 266-nanometer optical absorption peak of DNA, photoacoustic remote sensing is efficacious in recovering qualitatively similar nuclear information in comparison to that provided by the hematoxylin stain in the gold-standard hematoxylin and eosin (H&E) prepared samples. A photoacoustic remote sensing system was employed utilizing a 266-nanometer pulsed excitation beam to induce photoacoustic pressures within the sample resulting in refractive index modulation of the optical absorber. A 1310-nanometer continuous-wave interrogation beam detects these perturbed regions as back reflected intensity variations due to the changes in the local optical properties. Using this technique, clinically useful histologic images of human tissue samples including breast cancer (invasive ductal carcinoma), tonsil, gastrointestinal, and pancreatic tissue images were formed. These were qualitatively comparable to standard H&E prepared samples.

## Introduction

Histologic assessment of formalin-fixed, paraffin-embedded tissue remains the gold standard for clinical examination of resected tissue, cancer diagnosis, and assessment of surgical margins. The tissue preparation for clinical histopathological analysis is a resource intensive and time-consuming process involving fixation, processing, sectioning, and staining. The turnaround time for diagnosis can vary considerably between hospitals, complexity of the specimen and the type of cancer, with some taking as long as three days or more to render diagnosis^1^. It is not yet feasible to accurately distinguish between malignant and healthy tissue with intraoperative visual assessment of the surgical field which can lead to variability in surgical outcomes^2,3^. To examine surgical margins intraoperatively, surgical oncologists rely on clinical judgment and pathologist assessment which might use frozen section histopathology analysis. Although intraoperative frozen histopathology is a powerful tool that decreases positive margin rates and subsequent surgeries, the accuracy of the frozen tissue section analysis compared to final pathologic analysis can be variable due to frozen tissue artefacts which deteriorate the tissue architecture rendering histological interpretation more difficult^4^. Furthermore, each cycle of frozen pathologic assessment takes up to one hour, which significantly prolongs operating time, cost, and increases anesthesia risks for the patient^5^.

New intraoperative imaging techniques are being developed to provide an alternative to intraoperative histopathology methods. Previous studies have used light sheet microscopy^6^, fluorescence nonlinear microscopy^7–10^, microscopy with ultraviolet surface excitation (MUSE) microscopy^11^, and structured light microscopy^12,13^ to recreate hematoxylin and eosin (H&E) histology images. However, these techniques may require the addition of fluorescent dyes or optical clearing of the sample for imaging. These labelling methods add logistic issues during an operation and introduce potential toxicities depending on the agent^14,15^. Label-free approaches have been presented with stimulated Raman scattering microscopy^16^. However, this technique has only been demonstrated with transmission-mode imaging which also requires thin samples and a complex and expensive picosecond scale (2 ps) dual pulse excitation.

Photoacoustic (PA) techniques take advantage of the large endogenous optical absorption contrast present in tissues. As a nanosecond timescale pulse is absorbed by a chromophore it causes a sudden change in temperature. This sudden change in temperature leads to transient thermoelastic expansion which in-turn generates acoustic waves in the ultrasound range. Furthermore, biological tissue exhibits excellent specificity in optical absorption curves enabling rich tissue differentiation^17–19^. For visualization of cell morphology, the ultraviolet (UV) absorption peak of DNA is a powerful target. Conventional ultraviolet photoacoustic microscopy (UV-PAM) techniques have demonstrated efficacious label-free visualization of cellular structure in tissue samples^20–22^. Indeed, some recent works have provided compelling histological imaging quality. However, UV-PAM methods require physical contact with the tissue using an acoustic coupling medium, which may be impractical due to the constrained operational working space, increased risk of infection, and requires expensive pre- and post-surgical sterilization. Also, in some UV-PAM devices the sample and portions of the apparatus were fully submerged in a water tank^23^.

There remains an unmet need for label-free cellular-scale imaging capable of providing real-time cellular structure visualization of optically thick (much thicker than transport mean free path) samples. Thick samples require a reflection-mode apparatus which is more appropriate for clinical *in-situ* applications. In this paper, we present an all-optical, fast, deep, label-free, non-contact, cellular-resolution, reflection-based optical imaging technique using the recently reported photoacoustic remote sensing (PARS) microscopy^24–26^. A salient feature which sets PARS apart from conventional PA modalities is that it operates fully non-contact, even demonstrating centimeter scale working distances. This permits optical absorption contrast to be visualised in turbid media without the need for acoustic coupling such as water or ultrasound gel. The goal with this technique is to extract diagnostic quality histopathological information.

In PARS a nanosecond excitation beam is co-focused with a continuous-wave probe beam into the target. The absorbed optical energy from the excitation pulse is converted to pressure through thermo-elastic expansion. This pressure rise produces elasto-optic modulations within the absorber, changing the intensity of the back-reflected probe beam. The magnitude of these optical signals is proportional to the optical absorption of the excitation wavelength^27,28^. Utilizing the UV absorption peak of DNA, the cell nuclei and bulk tissue structure can be visualized. Employing PARS detection, we present the first label-free non-contact histology-like images of breast, gastrointestinal, and skin tissues from formalin-fixed paraffin embedded (FFPE) blocks and unstained thin slices. These images are qualitatively compared by imaging co-localized regions and quantitatively compared by several nuclear morphology metrics to conventional H&E prepared slides to evaluate the diagnostic potential of this approach.

## Methods

### Experimental Apparatus

**Figure 1:**
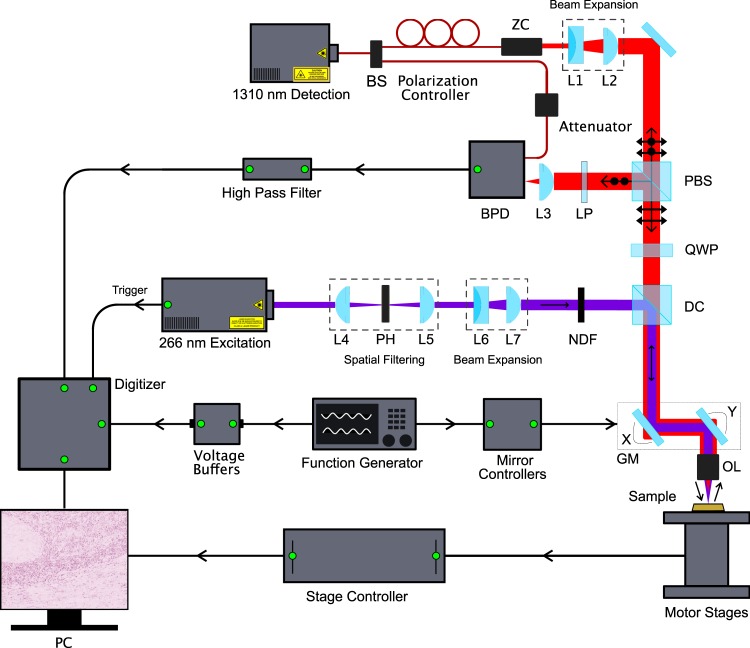
Schematic of the PARS apparatus. Component labels are defined as: beam splitter (BS), zoom collimator (ZC) long-pass filter (LP), polarizing beam splitter (PBS), quarter-wave plate (QWP), dichroic mirror (DC), balanced photodiode (BPD), pinhole (PH), neutral density filter (NDF), galvanometer mirrors (GM), objective lens (OL). The 266 nm laser (SNU-20F-10x, Teem Photonics) was spatially filtered by two lenses (LA4052-UV and LA4380-UV, Thorlabs Inc.) and a 25 µm pinhole (P25C, Thorlabs Inc.). The beam was then expanded using a fixed magnification beam expander (BE05-266, Thorlabs Inc.). This was combined with the detection path which uses a 1310 nm continuous-wave superluminescent diode (S5FC1018P. Thorlabs Inc.) as a source. The 1310 nm beam was passed through a fiber polarization controller (FPC562, Thorlabs Inc.) and then collimated (RC04APC-P01, Thorlabs Inc.). The collimated interrogation beam was then expanded using a variable beam expander (BE02-05-C, Thorlabs Inc.). The polarizing beam splitter (CCM1-PBS254, Thorlabs Inc.) splits the beam based on its polarization state. The horizontally polarized light is converted to circularly polarized light using a zero-order quarter wave plate (WPQ10M-1310, Thorlabs Inc.). A dichroic mirror (HBSY234, Thorlabs Inc.) is used to combine the detection beam and the excitation beam. The excitation and detection beams were co-aligned into a 2D galvanometer scanning mirror system (GVS412, Thorlabs Inc.). The beams were then co-focused using a 0.3 numerical-aperture reflective objective lens (LMM-15X-UVV, Thorlabs Inc.). The back-reflected interrogation beam was converted from circular to horizontal polarization by the quarter waveplate and then passes back through the polarizing beam splitter where it is directed to a 75-MHz bandwidth InGaS balanced photodiode (PDB425C-AC, Thorlabs Inc.) for detection. The long-pass filter is chosen to block any back-reflected excitation light.

The PARS microscope used in these experiments (as shown in Fig. 1) consists of two optical beams. The excitation beam, which provides optical absorption contrast, comes from a 0.5 ns pulsed 266 nm source while the detection beam is provided by a 1310 nm continuous-wave superluminescent diode. The 266 nm wavelength of the pulsed excitation primarily targets a DNA optical absorption peak. Upon absorption of a pulse, thermoelastic expansion within the cell nuclei creates large initial photoacoustic pressures. These pressures can modulate the local optical properties through the elasto-optic effect. This creates a perturbed optical scattering profile seen by the detection beam. If the cell nuclei are considered to be an optically scattering particle with the cytoplasm of the cell, then these elasto-optic modulations modify these scattering properties. This scattering perturbation can then be observed with the probe beam as a rapidly changing back-scattered intensity coincident with the excitation spot. Although the elasto-optic effect itself produces a relatively small change in the optical properties (such as the permeability and permittivity) this effect is amplified by the pre-existing scattering profile which exists between the cell nuclei and cytoplasm. Additional details have been explored in previous works^24–28^.

### Image formation

Small field of view images, typically less than 300 µm wide, were formed by optically scanning over the target tissue using a 2D galvanometer mirror system. The mirrors are mounted at right angles to each other and therefore serve to steer the beam to any point via optical scanning on the focal plane through the main objective. One of the mirrors is operated at a faster frequency, typically 30 Hz, whereas the frequency at which the slower mirror operates is determined by the point density required and the laser repetition rate. As an example, for a 400k point scan, a 60 mHz frequency is adequate. The acquisition time is solely dependent on the point density at a given excitation repetition rate, with the 400k point scan taking 18–20 s per frame.

The voltage signal from the balanced photodiode is fed to a 16-bit digitizer card (CSE161G4, Gage Applied). The PARS signals are then extracted by taking the envelope of the recorded time domain signals indicative of the PARS modulations within the sample. The images are formed by the taking maximum amplitude projection at each point, forming a C-scan *en-face* projection. Positional data from the scanning mirrors is also acquired and averaged to obtain more accurate positions. To render the raw data in a Cartesian grid, Delaunay interpolation is performed on the signals.

Large field of views are formed by capturing mosaics with a combination of linear translation stages (XMS-100S, Newport, Inc.) and mirror scans. The stages move the sample in a grid pattern. This pattern is computed by an in-house developed software which also controls the acquisition and moves the samples underneath the lens. At each point in the grid, a mirror scan is acquired and saved to disk. Volumetric scans are acquired by moving the sample with a vertical stage (GTS30V, Newport, Inc). The frames for volumetric scans are also acquired using the scanning mirrors. The aforementioned software controls the vertical stage and acquires an image at designated offsets. The acquired images from both the mosaic and volumetric scans are reconstructed using an in-house MATLAB script to perform interpolation. The mosaic frames are assembled together using Image J’s Grid/Collection Stitching Plugin^29^, which measures the cross-correlation between adjacent images and finds the best position for overlaying them.

The mirror scans form the basis for the mosaics and volumetric scans presented in the paper and serve as an imaging mode which allows for zooming into an interesting region of a large field of view image. To acquire these large field of view scans the scanning mirrors are held stationary and the sample is moved via the mechanical stages under the objective. Mechanical position feedback is recorded for reconstruction. This imaging mode allows for essentially unlimited field of view, with the primary limitations being point density and acquisition time. For example, large scans were commonly performed at 10 × 10 mm with a 4 µm resolution, in which the acquisition time was around 7 minutes.

### Resolution study

**Figure 2:**
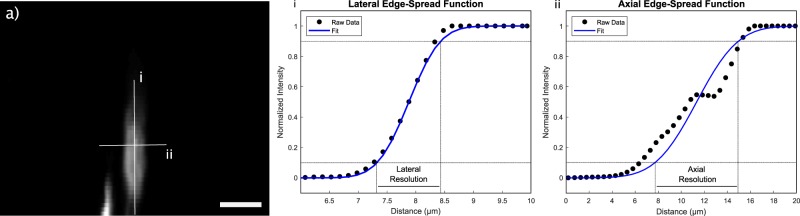
Resolution study from a 7 *μ*m diameter carbon fiber in water. (**a**) A B-Scan of the carbon fiber is shown. The two right graphs show how the lateral and axial resolutions are extracted from edge spread functions of the carbon fiber (i) and (ii) respectively. Scale bar 10 µm.

The system resolution was characterized against a B-scan of a carbon fiber. The lateral and axial resolution is measured by fitting raw data of the B-scan to edge spread functions. The resolution is defined as the width from 10% to 90% of this function as illustrated in Fig. 2. The lateral resolution of the system is found to be 1.2 µm whereas the axial resolution is measured at 7.3 µm. This resolution can be compared to commercial bright-field microscopes used in pathology which generally operate with objectives providing 5x to 40x magnifications yielding submicron resolutions.

### Sensitivity characterization

Signal to noise (SNR) values are performed by first characterization of the system noise. The noise (*σ*_*n*_) is characterized as the standard deviation of all pixel values in empty frames. Then the SNR measurements were performed with two metrics. The peak SNR (*SNR*_*p*_) and mean SNR (*SNR*_*m*_) are taken as

$$SN{R}_{p}=\frac{max\{|{s}_{ij}|\}}{{\sigma }_{n}}$$

$$SN{R}_{m}=\frac{{|{s}_{ij}|}_{{s}_{ij} > t}}{{\sigma }_{n}}$$

respectively where *s*_*ij*_ is the signal in the image, 〈〉 represents a mean, and *t* is some threshold value used to denote signal level. In tissue samples, this is the maximum value at which there is reasonable structural recovery of nuclear structures. Extracted SNR values follow as *SNR*_*p*_ = 58.45 dB and *SNR*_*m*_ = 41.98dB.

### Sample preparation

Human breast, tonsil, gastrointestinal and pancreatic tissue samples were obtained under a protocol approved by Research Ethics Board of Alberta (Protocol ID: HREBA.CC-18-0277) and University of Waterloo Health Research Ethics Committee (Humans: #40275 Photoacoustic Remote Sensing (PARS) Microscopy of Surgical Resection, Needle Biopsy, and Pathology Specimens). All experiments were performed in accordance the relevant guidelines and regulations. Tissue specimens were attained from anonymous patient donors with the help of clinical collaborators in the Cross Cancer Institute (Edmonton, Alberta, Canada). The ethics committees waived the requirement for patient consent on the condition that samples were archival tissue no longer required for diagnostic purposes, and that no patient identifiers were provided to the researchers. The breast tissue specimens were obtained from fresh mastectomy resections, and immediately placed in formaldehyde to allow for proper tissue fixation. The pancreatic tissue and tonsil resection tissue were obtained from stored samples that were prepared in a similar fashion.

To enable comparisons between PARS images and H&E stained images, adjacent sections were cut from the same FFPE tissue block. As they are not the same exact tissue sample, some minor differences in appearance do exist. The method of preparation for the tissue blocks, unstained slides and H&E slides follows.

### Tissue blocks

After remaining submersed in formaldehyde for at least 48 hours, the tissues were then processed as usual: dehydration, cleared with xylene and infiltrated with hot paraffin wax. Thereafter the tissue was embedded in paraffin wax and mounted on cassettes. Once cooled to room temperature, the FFPE blocks were finally created. This FFPE block could then be cut using a microtome. It was shaved down until the desired target tissue region was exposed. The gastrointestinal tissue was obtained from stored FFPE block samples that were initially prepared in a similar fashion.

### Unstained slide preparation

4 µm thick tissue slices from the FFPE blocks were acquired and the resulting ribbons were placed in a warm water bath. The ribbons were then transferred to glass slides and baked at a temperature of 60 °C for one hour in order to remove the excess paraffin. The slides were not covered with coverslips or any other media.

### H&E slide preparation

4 µm thick tissue slices from the FFPE blocks were acquired and the resulting ribbons were placed in a warm water bath. The ribbons were then transferred to glass slides and baked at a temperature of 60 °C for 30 minutes. The slides were then stained with hematoxylin and eosin staining media before covering with mounting media and a coverslip. Once the mounting media was dry, the slides were considered ready to use.

## Results

### System Characterization

**Figure 3:**
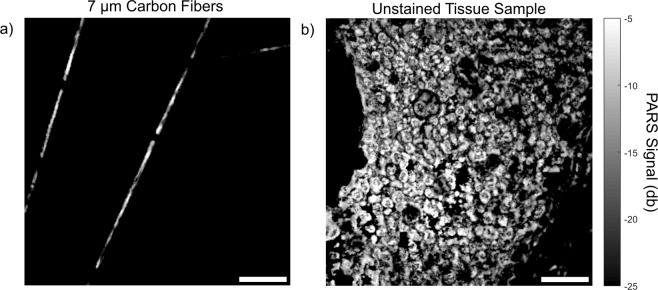
(**a**) PARS image of 7 µm carbon fibers. (**b**) PARS images of an unstained slide-mounted breast tissue sample. Scale bars for (**a**,**b**) are 50 µm.

Results are acquired using the apparatus detailed in Fig. 1. In brief, a 266 nm nanosecond excitation pulse is used to excite photoacoustic pressures within the sample providing DNA contrast. These pressures are detected by monitoring the back-reflected intensity of a co-focused 1310 nm continuous-wave interrogation source. Targets include 7 µm diameter carbon fiber networks (Fig. 3a) and breast cancer tissue (invasive ductal carcinoma) (Fig. 3b). All images are acquired with endogenous contrast and without additional labels. All PARS images presented were acquired with a large working distance from the objective (24 mm) facilitating implementation both as a table-top device, and as an *in-situ* tool where working space is limited. With the current pulsed repetition rate of 20 kHz, small field of view (high resolution) acquisitions are acquired at a point density of 2 pixel/µm and at a rate of 1250/*s*. Resolution metrics are pulled from structural images by using edge spread functions across B-scans of 7 µm carbon fibers. The lateral resolution is found to be 1.2 µm while the axial resolution is measured at 7.3 µm. Signal to noise (SNR) values are performed by first characterizing the system noise by imaging blank frames, then comparing against PARS signals. This yields a peak and mean SNR of *SNR*_*p*_ = 58.5 dB and *SNR*_*m*_ = 42dB respectively in unstained tissue slides (Fig. 3b). Further details regarding these measurements are provided in the Methods section.

### Assessment of human tissue

**Figure 4:**
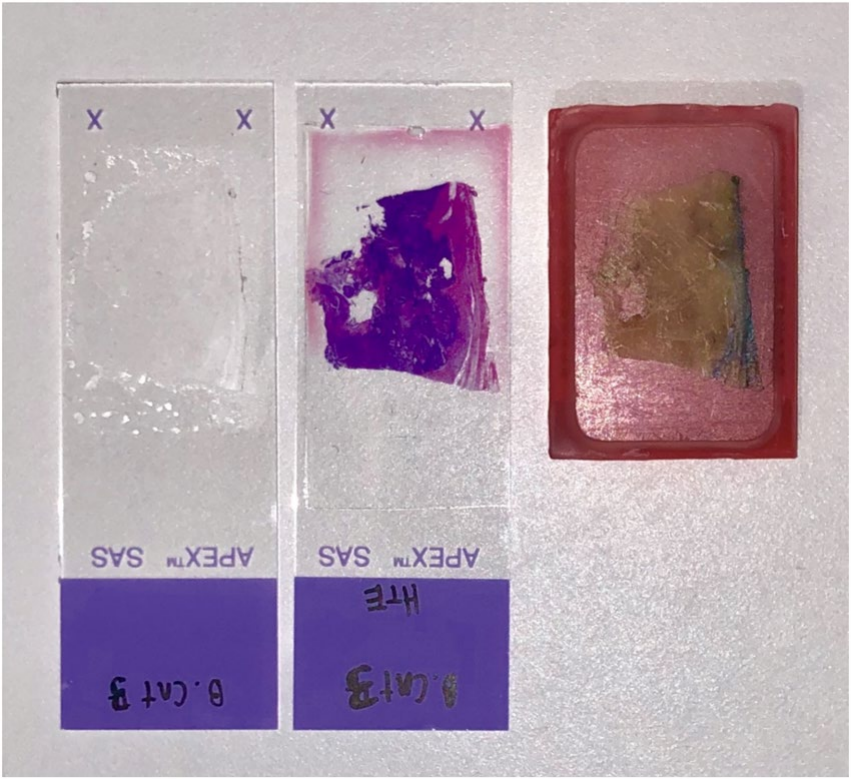
A comparison of the three sample types discussed in this work: (left) a formalin-fixed paraffin-embedded tissue block; (middle) a conventional H&E prepared slide and; (right) an unstained tissue slide.

**Figure 5:**
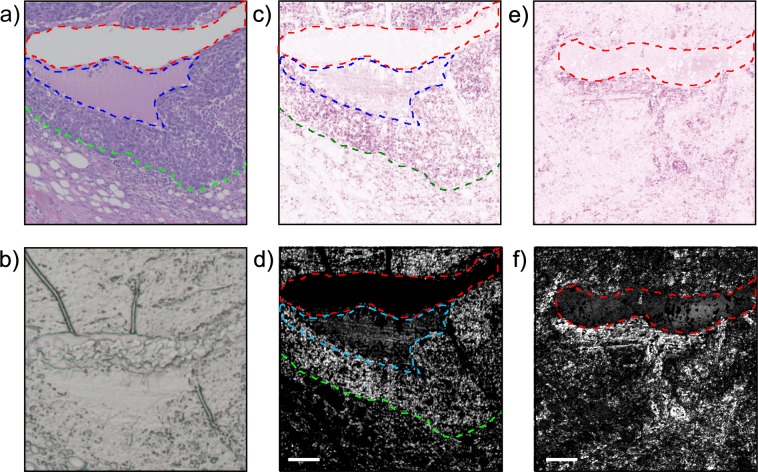
A comparison between a PARS images and (**a**) a bright field image at the of the same breast tissue sample with invasive ductal carcinoma. (**b**) an unstained slide imaged with a conventional bright field microscope. A PARS acquisition of the regions is shown for the unstained tissue slide with (**c**) a false H&E colour map applied and a (**d**) logarithmic greyscale colour map applied. A PARS acquisition of a FFPE block with (**e**) a false H&E colour map applied and (**f**) a logarithmic greyscale colour map applied. Green outline highlights a sharp delineation between a hypercellular region of tissue and healthy tissue, blue outline represents a high-grade malignancy with serosity and necrotic cells, the red outline represents an artifact present in the slides. Scale bar 100 µm.

**Figure 6:**
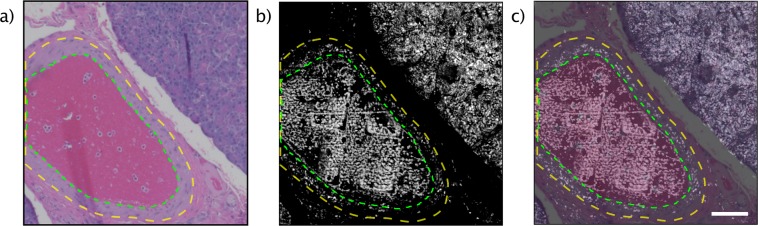
(**a**) A standard H&E prepared slide imaged with a standard pathology microscope of a blood vessel (within green outline) located in pancreatic tissue, surrounded by muscular layer (between green and yellow outline) with smooth muscle cells. (**b**) An unstained tissue slide of the adjacent section imaged with PARS. (**c**) A direct overlay of the images (**a**,**b**). Scale bar 100 µm.

**Figure 7:**
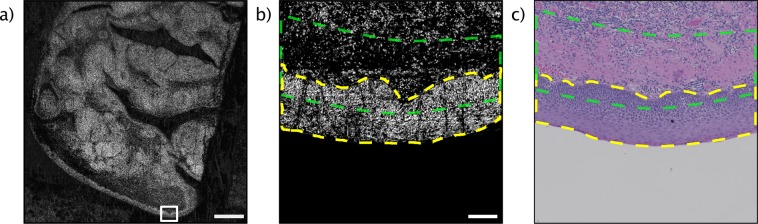
(**a**) A large field-of-view scan of a tonsil tissue specimen using PARS microscopy. Scale bar 1 mm. (**b**) A high resolution zoomed-in scan of the skin margin its superficial hypercellular stratified squamous epithelium (yellow outline) and the sharp delineation with the underneath hypocellular stroma (green outline) using PARS microscopy. Scale bar 100 µm. (**c**) an H&E prepared tissue slice of the adjacent section of tonsil tissue.

Figure 4 compares the three samples types examined in this study. Two preparations are used for the PARS samples including unstained glass slide-mounted sections and FFPE blocks. Consecutive tissue sections of breast tissue were prepared as unstained FFPE blocks and unstained slide-mounted sections along with a conventional H&E preparation. To approximate the surface of bulk unprocessed tissue, the unstained slide-mounted samples are not protected by a coverslip. These tissue samples provide a uniform control target to avoid out-of-focus artifacts from optical sectioning. The FFPE blocks more closely approach *in-situ* targets in that they contain tissue which can only be captured *en-face* with a reflection-mode modality and provide a multilayered structure for volumetric acquisitions. These tissue samples are roughly 5 mm thick, are embedded in paraffin, and mounted on an opaque embedding cassette. In addition, the FFPE blocks have micron scale surface irregularities similar to fresh tissue. The unstained samples are imaged using the PARS apparatus detailed in Fig. 1 whereas the H&E images are acquired using a standard conventional bright field pathology microscope (Zeiss Axioscope 2 with Zeiss Axiocam HR). The performance of PARS in visualizing cellular-scale structures is compared to conventional H&E bright field microscopy using breast (Fig. 5), gastrointestinal (Fig. 6) and tonsil tissues (Fig. 7). Similar regions are imaged across all three sample types for direct comparison.

Figure 5a is a standard H&E stained image taken with a conventional bright field microscope at a magnification of 5x. The unstained tissue slides were also imaged with a bright field microscope for comparison (Fig. 5b). PARS images with false H&E colour map (Fig. 5c,e) is used to approximate a hematoxylin-like stain as seen in the H&E stained bright field images, and a greyscale logarithmic colour map (Fig. 5d,f) is used to highlight the PARS signal. In the false H&E colour map, darker pinks represent stronger PARS signals which is primarily attributed to higher DNA concentrations in these locations where the white indicates minimal PARS signal and a lack of DNA contrast. In the greyscale colour map white represents stronger PARS signals where black is associated with weaker PARS signal. However, as the primary contrast mechanism is the DNA absorption peak, direct visualization of eosin-stained regions is not achieved.

Other examples of human tissue samples are shown in Figs 6 and 7 where pancreas and tonsil tissues are presented. The brightfield images (Figs 6a, 7c) are captured at a magnification of 10x and 20x, respectively. Each is compared with a conventional H&E image of the consecutive layer. As well, Fig. 7a highlights the wide field of view (1 cm by 1 cm) imaging capabilities of the system. Additional acquisition details are provided in the Methods section.

### Tissue volumes

**Figure 8:**
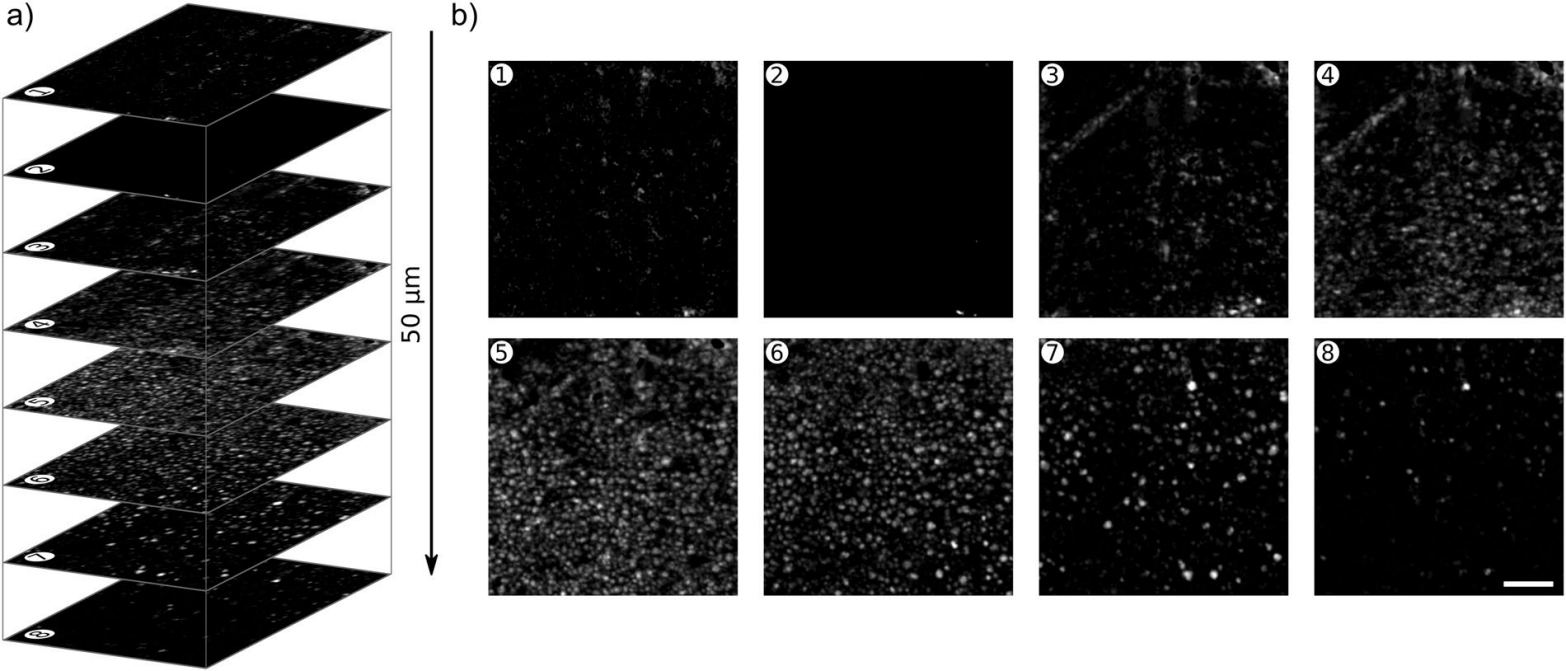
A volumetric scan of breast cancer tissue (invasive ductal carcinoma) (**a**) Consecutive slices are shown with separation of 7 µm. (**b**) The sections from (**a**) are shown. Scale bars 20 µm.

Volumetric scans were performed on the FFPE blocks to image multiple cell layers (Fig. 8). Volumes are acquired by taking PARS images at 100 depth levels in the sample at 500 nm steps. For the volume shown in Fig. 8 the total scan time was around 33 minutes giving an acquisition rate of about 360 *μm*^3^/*s*. Several distinct cellular layers are recovered at various depths and cellular resolution is maintained ~50 µm down into the sample. Further details can be found in the Methods section.

### Internuclear distance and cross-sectional area comparison

**Figure 9:**
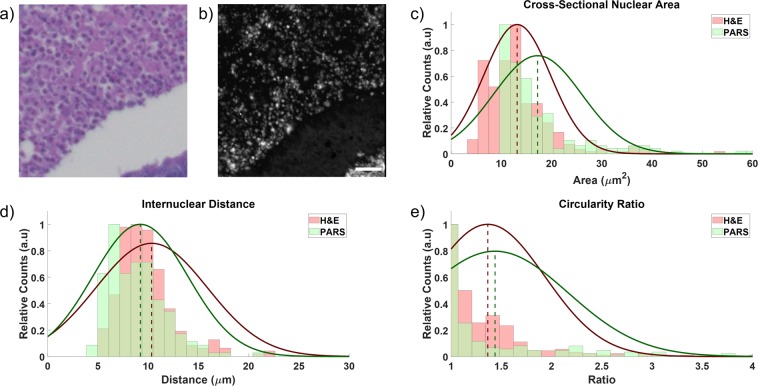
Quantitative study from (**a**) an image of a standard H&E prepared breast tissue sample captured by a commercial bright field microscope (**b**) a PARS image of the adjacent breast tissue section on the FFPE block. (**c**) A comparison of the cross-sectional nuclear area, (**d**) a comparison of the internuclear distance between cell nuclei, and (**e**) a comparison of the circularity ratio extracted from images (**a**,**b**). See Table 1 for further details.

PARS acquisitions (Fig. 9a) were compared to the adjacent H&E image (Fig. 9b) with several quantitative metrics. The cross-sectional area of the cell nuclei (Fig. 9c), the internuclear distance (Fig. 9d), and a circularity ratio of the cell nuclei (Fig. 9e) are shown with results summarized in Table 1. Three quantitative metrics are used to compare the bright-field H&E images with the PARS acquisitions. This is the internuclear spacing, nuclear cross-section, and the circularity of the nuclei. Internuclear spacing and nuclear cross-section are first pulled from images by thresholding nuclear structures from the background. The center of mass of each nucleus is determined by arithmetic mean along each dimension. Then, the minimum calculated distance between each centre of mass is determined to be the reported internuclear spacing. The nuclear cross-section is found for each nucleus by summing the total number of constituent pixels and multiplying by the area of each pixel. The circularity ratio is defined as $CR=\frac{{C}^{2}}{4\pi A}$ where *C* and *A* are the circumference and area of each cell nuclei respectively. If the nuclei are perfectly circular, this ratio would be unity. Deviation from unity suggests a non-circular shape. The perimeter of each object is taken as the sum of perimeter pixels times the dimension of each pixel, and the area is as defined for the cross-section. Diagnostically relevant information of tissue types may be pulled from mean values whereas cancerous tissues may exhibit larger variances.

**Table 1:** The calculated mean, standard deviation (SD) and overlap coefficients for the 3 qualitative analysis techniques for the H&E and PARS images in Figs. 9a,b.

Summary of Cell Morphology Metrics									
	Cross-Sectional Nuclear Area			Internuclear Distance			Circularity Ratio		
	Mean	SD	Overlap	Mean	SD	Overlap	Mean	SD	Overlap
H&E	13.3	7.7	0.79	10.3	5.6	0.89	1.42	0.77	0.87
PARS	17.5	9		9.21	4.7		1.54	1	

## Discussion

The system presented here highlight several important capabilities necessary for histology-like imaging of tissues. PARS does not require additional contrast to be added to the sample, the unstained slides provide a reasonable contrast-free analog to the prepared and stained H&E samples. The contrast in the PARS images is provided by the 266 nm absorption peak of the sample, primarily highlighting DNA concentration. However, other surrounding species are likely to contribute signal to some degree such as cytochrome, hemoglobin and collagen^30^ to name a few as they exhibit non-zero absorption at 266 nm. The high concentration of DNA in cell nuclei facilitates visualization of nuclear morphology, nuclear density, and cellular distribution. This is due to the PARS signal being proportional to the optical absorption which is in-turn proportional to the chromophore concentration. The contrast seen in H&E sections is from cell nuclei (purple) and the surrounding cytoplasm (pink) (Figs 5a, 6a, 7c). The hematoxylin dye is principally used as a nuclear stain whereas eosin visualizes the cytoplasm. The PARS images in this study provide contrast analogous to the hematoxylin stain as the 266 nm wavelength is strongly absorbed by DNA within cell nuclei. This relationship is similar to the one that exists in H&E preparation where the intensity of the hematoxylin stain is proportional to the negative ion concentration present in DNA and RNA. Since PARS operates in reflection-mode, the samples are not required to be thin, negating additional sectioning. Both capabilities are essential to achieve *in-situ* imaging capability and are demonstrated with the FFPE blocks.

The ability to image thick tissue combined with DNA’s strong absorption at 266 nm may also permit the imaging of freshly resected tissue. Freshly resected tissue can potentially contain blood and other fluids. The extra blood and serosity can be removed with absorbing paper before inking the specimen. This is standard practice and is routinely done before staining the tissue. However, this extra step may not be required as the excess fluids would not interfere with the system’s ability to visualize surgical margins. Although blood appears as an opaque fluid macroscopically, the majority of its contrast is exhibited by erythrocytes which are discrete cells. Thus, under magnification the extra contrast from hemoglobin does not disrupt histological imaging. With PARS, there is an added benefit that erythrocytes are anuclear and do not exhibit significant optical absorption as compared to DNA at 266 nm. Since DNA absorbs 266 nm wavelength significantly more than hemoglobin and other chromophores^30^, the dominant contrast would still be from DNA facilitating the visualization of surgical margins and cellular morphology. Similarly, this work can also be applied to surgical cavities left behind by resections. Such resections can extend deep into the body and the surgeon must proceed with hemostasis. Presently, the surgeon must palpate the cavity in order to identify residual nodularity. This work may be applied to such cavities to visualize any residual cancer. The non-contact operation of this modality also minimizes the increased risk of infection posed by palpating deep cavities in the body.

“PARS images of the unstained slides highlight salient features of the sample including regions of hypercellularity and distinct boundaries between different tissue types. Figure 5 shows bright-field images of H&E stained (Fig. 5a), unstained tissue slides (Fig. 5b), PARS images of the same unstained tissue slide (Fig. 5c,d) and FFPE blocks (Fig. 5e,f). The tissue sample consists of human breast tissue presenting invasive ductal carcinoma. The H&E image (Fig. 5a) highlights a sharp delineation between cancerous and healthy cells (green outline). A high-grade tumour (blue outline) is also seen in the same figure whereas the red outline accentuates a tissue processing artifact. In contrast, in the unstained bright field image (Fig. 5b), the high-grade tumour and the sharp delineation between diseased and healthy tissue are not visible. The PARS images (Fig. 5c,d) of the unstained tissue demonstrates the visual differences and morphology of the breast gland duct from exocrine and cancerous cells (green outline) and adjacent adipose tissue with comparable fidelity to the H&E image. The processing artifact and the high-grade tumour are also clearly visible in the PARS images of the unstained tissue slide. The PARS images of the FFPE blocks (Fig. 5e,f) are concordant with the co-localized unstained sectioned samples and the bright-field H&E images. Salient micron-scale structure such as hypercellularity, internuclear spacing, nuclear morphology, and intranuclear density are captured, along with larger-scale features such as ducts, adipose tissue, connective tissue, and blood vessels. Where the FFPE blocks provide a closer analog to *in-situ* tissues, the unstained sectioned slides yield a closer analog to the conventional H&E processed slides as the preparation procedure follows a similar process but lack the contrast dyes. Figure 6 cellular morphology in human pancreatic tissue with H&E stained and PARS images. Figure 6a is a standard H&E image that of a medium sized blood vessel that is surround by a muscular layer. Bulk tissue and cellular morphology are visualized in the PARS image (Fig. 6b) and found to be comparable to the H&E image. Due to presence of nucleated cells (smooth muscle cells) present in the muscular layer surrounding the blood vessel (between yellow and green outline). Figure 6c is an overlay of the H&E image and PARS image demonstrating the agreement between the two modalities. Figure 7 compares H&E images and PARS images of human tonsil tissue. Figure 7b demonstrates the difference between the hypercellular stratified squamous epithelium and the sharp delineation (yellow outline) with the underneath hypocellular stroma.”

In the future, a method is required to provide images with eosin-like cytoplasmic staining patterns, where co-imaging would provide the dual contrast signal analogous to H&E staining. PARS is capable of incorporating additional excitation wavelengths which could be added to provide contrast for additional chromophores, targeting various components of tissue structure. To achieve eosin-like contrast, a roughly 400 nm excitation wavelength has previously shown to reveal cytochromes in the cytoplasm^22^. An infrared excitation source such as 1210 nm or 1720 nm can also be added to extract contrast provided by adipocytes, which is otherwise unavailable in standard H&E preparation due to dissolution of lipid by ethanol and xylene during tissue processing^31^. This additional contrast may reduce ambiguity of the PARS chromophore recovery at any given wavelength. Nonetheless, nuclear features, and other tissue features such as blood vessels (Fig. 6), duct morphology, hypercellularity and connective tissue structures are readily evident on the PARS images of excised tissues. Future iterations can multiplex different excitation wavelengths targeting specific chromophores in the same acquisition session, which allows for large amounts of diagnostic information to be extracted quickly, label-free, and *in-situ*.

Volumetric imaging experiments (Fig. 8) highlight the performance of the apparatus in thick tissue samples. Here, imaging was performed across a depth range of 50 µm showing several cellular layers. This appears to be the penetration limit for the current system in these FFPE blocks and is similar to the depth reported in previous transmission-mode UV-PAM works^21^. Since the transport mean free path of the 266 nm ultraviolet light is on the order of ~100 µm in human tissue, this range may be expected for an optical system which operates at these wavelengths. PARS has previously demonstrated the ability to exceed the transport mean free path of the excitation wavelength in tissue using a deeper penetrating detection beam^25^. This may suggest appreciable interaction between the 1310 nm detection source and the FFPE blocks’ paraffin media. Future efforts may need to investigate other detection wavelengths for imaging such prepared samples.

The qualitative analysis techniques used may provide additional metrics for PARS-based computer-aided diagnostics which is essential for approaching the gold-standard of human pattern recognition. This is critical for tissue identification and initial tissue diagnostics. For instance, the variance in the cross-sectional area and circularity of cell nuclei is indicative of nuclear aberrancy and the internuclear distance can indicate hypercellularity, which could suggest the presence of cancerous tissue. The extracted values show good correspondence between the two datasets suggesting PARS is capable of accurate visualization of nuclear structure. This highlights the potential for PARS microscopy in computational diagnostics and especially surgical margin assessment.

The imaging characteristic demonstrated reasonably fast frame acquisition times and sub-cellular resolution. However, the current system would be incapable of delivering full-quality frames as shown at real-time rates (greater than a frame per second). Imaging speeds can be improved by increasing the pulse repetition rate of the laser, potentially achieving real-time imaging speeds. PARS microscopy has demonstrated higher imaging rates in comparison to contact-based photoacoustic imaging methods due to the optical detection. The rate of optical detection with PARS is limited by the local stress confinement rather than acoustic propagation. The method has previously demonstrated acquisition rates up to 5 MHz^32^ suggesting if an appropriately fast repetition rate excitation is implemented, real-time feedback to clinicians is likely feasible.

## Conclusion

In this work preliminary steps towards an *in-situ* imaging technique are achieved and live histology-like information was demonstrated. The reflection-mode operation enables the imaging of thick tissue samples and may enable recovery of cellular structure in freshly resected human tissue and *in-situ*, circumventing the time-consuming process of fixing tissue in FFPE blocks. The non-contact method avoids additional complications such as an increased risk of infection and avoids an expensive pre- and post-operative sterilization process. Additionally, the all-optical non-contact detection mechanism allows for visualizing absorption contrast without requirement of a bulky ultrasound transducer, which precludes clinical usage where there is a limited operational working space. This may permit future developments as a live intraoperative surgical microscope. Furthermore, the all-optical architecture may allow for simple integration with complimentary imaging modalities such as OCT, fluorescence microscopy, and other optical imaging techniques due to the lack of an ultrasound transducer and ultrasound coupling media. Several different human tissue samples including breast presenting invasive ductal carcinoma, tonsil, gastrointestinal, and pancreatic tissues were prepared and imaged in FFPE blocks and thin unstained slices. Subjective clinical opinion and qualitative analyses found these PARS images were to be in good correspondence with conventional H&E processed tissue slides from concurrent regions. Diagnostically relevant information such as nuclear morphology, internuclear distance and intranuclear density can be recovered without the need for exogenous labelling while in an all-optical modality which does not require physical coupling to the sample. Multiple cellular layers were visualized in bulk tissue samples. These capabilities display a positive step towards real-time *in-situ* recovery of tissue structure to aid in rapid clinical assessment of tissues.

## References

[1] Patel Samip, Smith Jennifer B., Kurbatova Ekaterina, Guarner Jeannette (2012). Factors that impact turnaround time of surgical pathology specimens in an academic institution. Human Pathology.

[2] Sabel Michael S., Rogers Kendra, Griffith Kent, Jagsi Reshma, Kleer Celina G., Diehl Kathleen A., Breslin Tara M., Cimmino Vincent M., Chang Alfred E., Newman Lisa A. (2009). Residual disease after re-excision lumpectomy for close margins. Journal of Surgical Oncology.

[3] McCahill Laurence E., Single Richard M., Aiello Bowles Erin J., Feigelson Heather S., James Ted A., Barney Tom, Engel Jessica M., Onitilo Adedayo A. (2012). Variability in Reexcision Following Breast Conservation Surgery. JAMA.

[4] Shi SR (2008). Evaluation of the value of frozen tissue section used as ‘gold standard’ for immunohistochemistry. Am. J. Clin. Pathol..

[5] Chang KH (2011). Novel 16-minute technique for evaluating melanoma resection margins during Mohs surgery. J. Am. Acad. Dermatol..

[6] Glaser, A. K. *et al*. Light-sheet microscopy for slide-free non-destructive pathology of large clinical specimens. *Nat. Biomed. Eng*., 10.1038/s41551-017-0084 (2017). 10.1038/s41551-017-0084 PMC5940348 29750130

[7] Cahill Lucas C, Giacomelli Michael G, Yoshitake Tadayuki, Vardeh Hilde, Faulkner-Jones Beverly E, Connolly James L, Sun Chi-Kuang, Fujimoto James G (2017). Rapid virtual hematoxylin and eosin histology of breast tissue specimens using a compact fluorescence nonlinear microscope. Laboratory Investigation.

[8] Tao Y. K., Shen D., Sheikine Y., Ahsen O. O., Wang H. H., Schmolze D. B., Johnson N. B., Brooker J. S., Cable A. E., Connolly J. L., Fujimoto J. G. (2014). Assessment of breast pathologies using nonlinear microscopy. Proceedings of the National Academy of Sciences.

[9] Tu Haohua, Liu Yuan, Turchinovich Dmitry, Marjanovic Marina, Lyngsø Jens K., Lægsgaard Jesper, Chaney Eric J., Zhao Youbo, You Sixian, Wilson William L., Xu Bingwei, Dantus Marcos, Boppart Stephen A. (2016). Stain-free histopathology by programmable supercontinuum pulses. Nature Photonics.

[10] Szu-Yu Chen, Shee-Uan Chen, Hai-Yin Wu, Wen-Jeng Lee, Yi-Hua Liao, Chi-Kuang Sun (2010). In Vivo Virtual Biopsy of Human Skin by Using Noninvasive Higher Harmonic Generation Microscopy. IEEE Journal of Selected Topics in Quantum Electronics.

[11] Fereidouni F (2017). Microscopy with ultraviolet surface excitation for rapid slide-free histology. Nat. Biomed. Eng..

[12] Schlichenmeyer Tyler C., Wang Mei, Elfer Katherine N., Brown J. Quincy (2014). Video-rate structured illumination microscopy for high-throughput imaging of large tissue areas. Biomedical Optics Express.

[13] Fu Henry L., Mueller Jenna L., Javid Melodi P., Mito Jeffrey K., Kirsch David G., Ramanujam Nimmi, Brown J. Quincy (2013). Optimization of a Widefield Structured Illumination Microscope for Non-Destructive Assessment and Quantification of Nuclear Features in Tumor Margins of a Primary Mouse Model of Sarcoma. PLoS ONE.

[14] te Velde E.A., Veerman Th., Subramaniam V., Ruers Th. (2010). The use of fluorescent dyes and probes in surgical oncology. European Journal of Surgical Oncology (EJSO).

[15] Zhu Dan, Larin Kirill V., Luo Qingming, Tuchin Valery V. (2013). Recent progress in tissue optical clearing. Laser & Photonics Reviews.

[16] Orringer, D. A. *et al*. Rapid intraoperative histology of unprocessed surgical specimens via fibre-laser-based stimulated Raman scattering microscopy. *Nat. Biomed. Eng*., 10.1038/s41551-016-0027 (2017). 10.1038/s41551-016-0027 PMC5612414 28955599

[17] Beard Paul (2011). Biomedical photoacoustic imaging. Interface Focus.

[18] Wang Lihong V., Gao Liang (2014). Photoacoustic Microscopy and Computed Tomography: From Bench to Bedside. Annual Review of Biomedical Engineering.

[19] Yao Junjie, Wang Lihong V. (2013). Photoacoustic microscopy. Laser & Photonics Reviews.

[20] Yao D-K (2012). Optimal ultraviolet wavelength for *in vivo* photoacoustic imaging of cell nuclei. J. Biomed. Opt..

[21] Wong Terence T. W., Zhang Ruiying, Hai Pengfei, Zhang Chi, Pleitez Miguel A., Aft Rebecca L., Novack Deborah V., Wang Lihong V. (2017). Fast label-free multilayered histology-like imaging of human breast cancer by photoacoustic microscopy. Science Advances.

[22] Zhang Chi, Zhang Yu Shrike, Yao Da-Kang, Xia Younan, Wang Lihong V. (2013). Label-free photoacoustic microscopy of cytochromes. Journal of Biomedical Optics.

[23] Wong, T. T. W. *et al*. Label-free automated three-dimensional imaging of whole organs by microtomy-assisted photoacoustic microscopy. *Nat. Commun*., 10.1038/s41467-017-01649-3 (2017). 10.1038/s41467-017-01649-3 PMC5680318 29123109

[24] Hajireza Parsin, Shi Wei, Bell Kevan, Paproski Robert J, Zemp Roger J (2017). Non-interferometric photoacoustic remote sensing microscopy. Light: Science & Applications.

[25] Reza Parsin Haji, Bell Kevan, Shi Wei, Shapiro James, Zemp Roger J. (2018). Deep non-contact photoacoustic initial pressure imaging. Optica.

[26] Haven, N. J. M. *et al*. Label-free non-contact imaging of cell nuclei using ultraviolet photoacoustic remote sensing microscopy (Conference Presentation), 10.1117/12.2508036 (2019).

[27] Bell Kevan L., Hajireza Parsin, Shi Wei, Zemp Roger J. (2017). Temporal evolution of low-coherence reflectrometry signals in photoacoustic remote sensing microscopy. Applied Optics.

[28] Bell Kevan, Hajireza Parsin, Zemp Roger (2017). Scattering cross-sectional modulation in photoacoustic remote sensing microscopy. Optics Letters.

[29] Preibisch S, Saalfeld S, Tomancak P (2009). Globally optimal stitching of tiled 3D microscopic image acquisitions. Bioinformatics.

[30] Soltani Soheil, Ojaghi Ashkan, Robles Francisco E. (2019). Deep UV dispersion and absorption spectroscopy of biomolecules. Biomedical Optics Express.

[31] Buma Takashi, Conley Nicole C., Choi Sang Won (2017). Multispectral photoacoustic microscopy of lipids using a pulsed supercontinuum laser. Biomedical Optics Express.

[32] Snider, L., Reza, P. H., Zemp, R. J. & Bell, K. L. Toward wide-field high-speed photoacoustic remote sensing microscopy., 10.1117/12.2291036 (2018).

